# S100A9 Derived From Myeloma Associated Myeloid Cells Promotes TNFSF13B/TNFRSF13B-Dependent Proliferation and Survival of Myeloma Cells

**DOI:** 10.3389/fonc.2021.691705

**Published:** 2021-06-03

**Authors:** Lingzhang Meng, Qiang Tang, Jingjie Zhao, Zechen Wang, Liuzhi Wei, Qiuju Wei, Lianfei Yin, Shiguan Luo, Jian Song

**Affiliations:** ^1^ Department of Radiation Oncology, Renji Hospital, School of Medicine, Shanghai Jiao Tong University, Shanghai, China; ^2^ Center for Systemic Inflammation Research (CSIR), School of Preclinical Medicine, Youjiang Medical University for Nationalities, Baise, China; ^3^ Department of Burn Plastic and Wound Repair Surgery, Affiliated Hospital of Youjiang Medical University for Nationalities, Baise, China; ^4^ Life Science and Clinical Research Center, Affiliated Hospital of Youjiang Medical University for Nationalities, Baise, China; ^5^ College of Pharmacy, Youjiang Medical University for Nationalities, Baise, China; ^6^ School of Imaging, Youjiang Medical University for Nationalities, Baise, China; ^7^ Department of Cardiovascular Surgery, Affiliated Hospital of Youjiang Medical University for Nationalities, Baise, China

**Keywords:** S100A9, TNFSF13B, myeloma, scRNA seq, tumor associated myeloid cells

## Abstract

Multiple myeloma (MM) is a lethal hematological malignancy characterized by abundant myeloid cells in the microenvironment that fuel tumor progression. But the mechanism by which myeloid cells support myeloma cells has not been fully explored. We aimed to examine their effect on bone marrow cells of MM patients by scRNA-seq transcriptome analysis and reveal a high-resolution gene profile of myeloma cells and myeloma-associated myeloid cells. Based on correlation analysis of integrated scRNA-seq and bulk RNA-seq datasets from patients, we confirmed that myeloid-derived S100A9 was involved in TNFSF13B-dependent myeloma cell proliferation and survival. In the animal experiments, S100A9 was found to be critical for MM cell proliferation and survival *via* TNFSF13B production by myeloid cells, neutrophils, and macrophages. *In-vitro* analysis of patient primary myeloma cells further demonstrated that enhanced TNFSF13B signaling triggered the canonical NF-*κ*B pathway to boost tumor cell proliferation. All these results suggest that myeloid-derived S100A9 is required for TNFSF13B/TNFRSF13B-dependent cell-fate specification, which provides fresh insights into MM progression.

## Introduction

Multiple myeloma (MM) is a lethal hematological tumor resulting from the uncontrollable proliferation of plasma cells, which cover about 10% of hematological malignancies (1). Because of an insufficient understanding of the roles of components within the tumor microenvironment (TME) in MM pathogenesis, personalized cancer medicine based on risk stratification for better prognosis has yet to be developed. Pre-existing studies of TME-associated plasma cells have identified the peripheral blood neutrophil (N) to lymphocyte (L) ratio (NLR) as a strong indicator for a poor prognosis (2). Tumor-associated macrophages (TAMs) are other key players responsible for tumor cell proliferation and survival, as will be described below (3). Among the abundant myeloid cells present in MM, whether neutrophils and macrophages have a prognostic impact on malignant progression remains to be explored. We are also curious to know which molecules are responsible for the activation of neutrophil/macrophage function in MM.

Toll-like receptors (TLRs) expressed on the surface of terminally differentiated plasma cells can recognize danger-associated molecular patterns (DAMPs), thus upregulating Blimp-1 and XBP-1 at the transcriptional level, which is critical for malignant plasma cell survival (4, 5). B cell-helper neutrophils are known to induce plasma cell formation and antibody production (6). A possible mechanism is that DAMP–TLR interaction induces S100 calcium-binding protein A9 (S100A9) secretion from neutrophils (7). S100A9 serves as a significant DAMP signal transducer and an endogenous ligand of TLR-4. It acts synergistically with TNFSF13B in producing pro-inflammatory autoantibody isotypes *via* the TLR-associated signaling adaptor MyD88 (8, 9). Dysfunctional TLR signaling leads to overproduction of type I interferons and uncontrollable growth of MM cells (10). Given the importance of TLR signaling for myeloma cells, it is unknown why the association of S100A9 with TLR activation, particularly their effects on the tumor cell fate, in myelomagenesis remains unexplored.

Belonging to the TNF superfamily, TNFSF13B confers survival signaling to B cells *via* binding to the two receptors, TNFRSF13B and TNFRSF13C, which involve the activation of non-canonical NF-*κ*B pathways. TNFSF13B delivers signals to plasma cells by interaction with TNFRSF13B and TNFRSF17, stimulating the MAPK/p38 pathway (11). In contrast to TNFRSF13C binding that leads to non-canonical NF-*κ*B pathways, such as those contained p38MAPK or the NF-*κ*B-p65 complex, TNFRSF13B binding is crucial for the activation of the canonical NF-*κ*B pathway (12, 13). Consistent with other observations, our previous study has recognized neutrophils as the major source of TNFSF13B in the spleen, which keep myeloma cells from apoptosis (14, 15). Notably, TNFSF13B has been proven to be associated with myeloma cell proliferation and myelomagenesis (16). It is worth taking a closer look at which TNFSF13B receptors and underlying signaling pathways determine MM cell fate.

In the current study, we aimed to provide more details on the role of myeloid-derived S100A9 in MM prognosis. Specifically, we examined the gene expression profile of myeloma cells and myeloma-associated myeloid (MAM) cells from monoclonal gammopathy of undetermined significance (MGUS) and MM patients using scRNA-seq transcriptome analysis. We found the correlation of myeloid-derived S100A9 with TNFSF13B dependency in myeloma cells. This finding was validated using a bulk RNA-seq dataset from 74 patients, and the correlation of TNFSF13B gene expression with patient survival was confirmed. In mice inoculated with myeloma cells and analysis of patient primary myeloma cells, S100A9 promoted myeloma cell proliferation and survival through TNFSF13B production by myeloid cells, and the activation of the canonical NF-*κ*B pathway was TNFSF13B/TNFRSF13B-dependent, as discussed later in this paper. All these provide improved knowledge of the pathogenesis of MM.

## Material and Methods

### Acquisition of Gene Expression Data

Due to the low incidence of MM, there were no relevant sample sets present in the tumor genome database (TCGA) (https://cancergenome.nih.gov/). We did the analysis using gene expression data from the GEO database (https://www. ncbi.nlm.nih.gov/gds/), including GSE104171 (total RNAseq data of 74 patient sample sets) (17) and GSE124310 (scRNA-seq data of 4,174 human bone marrow cell samples) (18).

### RNA-seq Bioinformatics Analysis

Using the two gene expression datasets, we calculated Pearson’s correlation coefficients between individual genes to identify genes intersected with S100A9 and TNFRSF13B. The correlation of TNFRSF13B and TLR4 mutation with disease progression was analyzed using cBioPortal (https://www.cbioportal.org/) (19). We also evaluated the correlation of TNFRSF13B and TNFRSF17 expressions with 3-year survival of MM patients recorded in the GSE9782 dataset using Oncomine (https://www.oncomine.org/) (20). Gene correlation was displayed using scatter plots or heatmap respectively.

### ScRNA Analysis

Integration of scRNA-seq data from different sources and processes was performed using the R package Seurat (version 4.0.0); data normalization and clustering were used to elucidate differentially expressed genes (21). Data from MGUS and MM samples were standardized with a sctransform method. We proceeded to perform principal components analysis (PCA) analysis on the integrated dataset, clustering the gene expression data at 0.1 resolution with Uniform Manifold Approximation and Projection (UMAP, version 0.2.6.0). Vlnplot was used to demonstrate the expression level within different UMAP clusters, and Featureplot projected the gene distribution and expression on the UMAP clusters. R package EnhancedVolcano was employed to visualize the differential expression between two clusters or subclusters.

### Culture of Human Monocytes

Human monocytes were isolated from a buffy coat. Cells were centrifugated on the Pancoll gradient (1.070 g/ml) and purified on the Percoll gradient (1.064 g/ml) to obtain the monocyte populations with a purity of >85%. Monocytes were cultured in McCoy medium (Sigma-Aldrich) with 15% FCS and 7% CO_2_, in the absence or presence of 5 mg/ml recombinant human S100A9 (9254-S9-050, R&D). TNFSF13B expression level in the monocytes was detected using Human TNFSF13B Quantikine ELISA Kit (R&D).

### Culture of Patient Myeloma Cells

Bone marrow cells were obtained from MM patients. Myeloma cells were isolated from bone marrow of MM patients using the Human Plasma Cell Isolation Kit (130-093-628, Miltenyi Biotech). The percentage of myeloma cells after purification was more than 90% as measured by FACS analysis. Cell culture was performed in RPMI 1640 medium with 10% FCS and 2 mM L-glutamine.

### Mice

The C57BL/6 mice used were 8–12 weeks old. All animal experiments were carried out according to the Chinese Animal Welfare Guidelines. Neutrophil and macrophage depletion and surface marker expressions of splenic cell populations were detected in mice.

### Neutrophil Depletion

Neutrophil depletion was performed by 100 μg anti-Ly6G intravenous injection (127602, BioLegend, 1A8). The efficiency of depletion of splenic resident neutrophils was verified by flow cytometry. Over 90% of splenic neutrophils were depleted 24 h after injection. An antibody rat IgG2a was used as isotype control, which showed a comparable neutrophil population to the untreated.

### Macrophage Depletion

Liposome containing clodronate (Liposome Flow) was administrated *via* intravenous injection (200 µl/mouse) to achieve sufficient macrophage depletion. We analyzed its influence on splenic macrophage B cells using FACS at the indicated time and found over 90% of splenic macrophages depleted within 48 h after injecting clodronate liposomes. In this experiment, control mice were administered with empty liposomes, in which the macrophage population was unchanged.

### S100A9 Inhibitor

Paquinimod (ABR-215757) is an immunomodulatory compound that prevents S100A9 from binding to TLR-4 (16). A 25 μg paquinimod (319595, MedKoo) dissolved in PBS (10 μg/g body weight) was injected intraperitoneally into mice once daily until the end of the experiment.

### Flow Cytometry Analysis

Single-cell suspension of murine spleens was obtained through a 70 μm cell strainer, and erythrocytes were removed with lysis reagent bought from BD Biosciences (349202). Surface markers of cells were analyzed on a FACSCalibur (BD Biosciences). Plasma cells (CD19^−^CD138^+^), neutrophils (Ly6G^hi^CD11b^hi^), and macrophages (CD45^+^F4/80^+^) were measured with intracellular staining with the following antibodies.

### Antibodies

Flow cytometry panels were built using the antibodies as follows: Biotin anti-CD23 (553139, Pharmingen), APC anti-Ly6G (127614, Biolegend), APC anti-CD11b (17-0112-83, eBioscience), FITC or PE anti-CD21 (552957, 553818, Pharmingen), PerCP anti- CD19 (552854, Pharmingen), APC rat anti- S100A9 (565833, BD Biosciences), and FITC anti-TNFRSF13C (11-5943, eBioscience).

To assess TNFSF13B signaling involved in MM, human myeloma cells were analyzed with a four-color immunophenotyping flow cytometry panel. The related antibodies used were as follows: APC anti-human CD138 (352308, Biolegend), PE anti-human TNFRSF13B (311906, Biolegend), PerCP anti-human TNFRSF17 (357509, Biolegend), FITC anti-human KI67 (151211, Biolegend), PE anti-human phospho-NF-*κ*B p65 (12-9863, eBioscience), and PE anti-human phospho-p38 (690203, Biolegend).

### Statistical Analysis

Quantitative variables were expressed as means ± standard deviation (SD). The significance of antibody titers, surface marker expressions, and splenic cell populations was analyzed with the unpaired Student *t*-test (GraphPad Prism). A *p*-value <0.05 was considered statistically significant.

## Results

**Figure 1:**
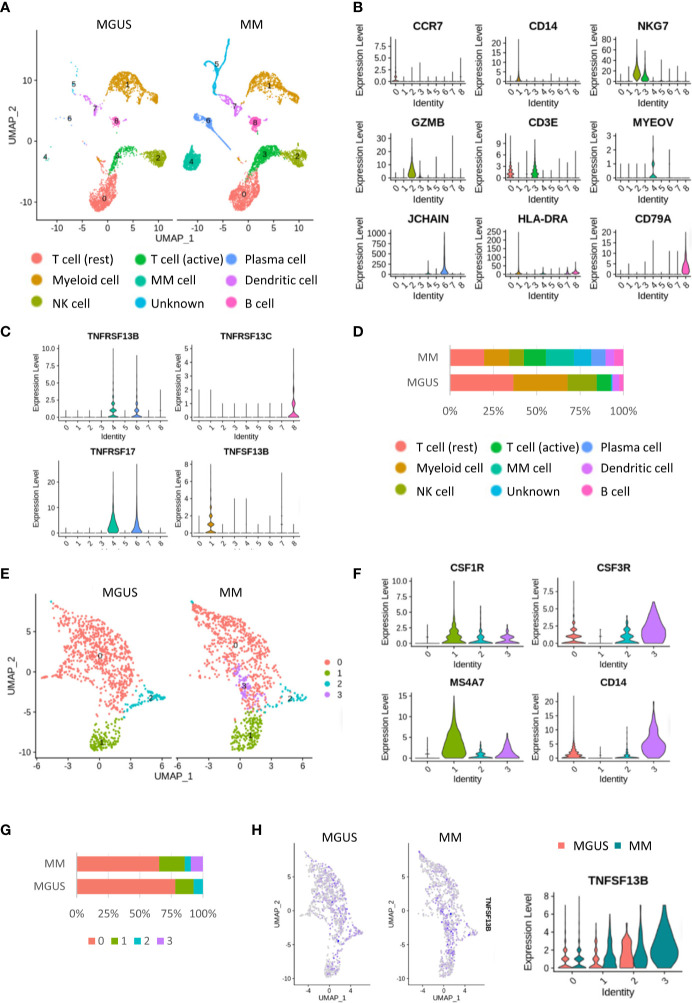
Single cell RNA-seq analysis in the MGUS and MM sample. **(A)** UMAP diagram shows the nine main cell types in the MGUS and MM samples. **(B)** Violin plots indicate the expression and distribution of marker genes in T cells, myeloid cells, NK cells, DCs, plasma cells, myeloma cells, and B cells. **(C)** Violon plots show the expression of TNFSF13B and its receptors on the plasma cell and myeloma cells. **(D)** In bar charts, the proportion of single cell data of MGUS and MM sample patients is quantified and compared. **(E)** UMAP diagram shows the subclustering of myeloid cells. **(F)** Violin plot indicates the different genes of neutrophil and macrophages. **(G)** In bar charts, the proportion of neutrophil and macrophages in the MGUS and MM sample is quantified and compared. **(H)** Feature plots demonstrate the projection of TNFSF13B expression onto the neutrophil and macrophages. Violin plots indicate the levels of TNFSF13B expression in the myeloid cells of MGUS and MM samples.

Single-cell sequencing (scRNA-seq) enables a faster understanding of the transcriptome profile of myeloma cells and their interactions with bystander cells in the TME. We constructed the landscape of cells in the MM microenvironment using the scRNA-seq dataset (GSE124310). We made the unsupervised dimensionality reduction and hierarchical clustering analysis and found nine main cell clusterings: T cells (c0, 3), myeloid cells (c1), myeloma cells (c4), NK cells(c2), plasma cells(c6), DCs (c7), and B cells (c8) (**Figures 1A–C** **)**. Each cell cluster was carefully annotated based on different expressions of known marker genes and distinctly identified from MGUS and MM samples (**Supplementary Figure 1**). Compared to the MGUS, MM patients were characterized by an evident rise in MYEOV+ myeloma cells, in which a clear upregulation of TNFSF13B receptors, TNFRSF13B and TNFRSF17, was detected (**Figures 1C, D** **)**. Another receptor, TNFRSF13C, mainly expressed in B cells, is correlated with the non-MM situation and indicates the irrelevance of TNFRSF13C for myeloma cells. Since the ligand TNFSF13B was produced by myeloid cells, we performed a myeloid cell subset analysis, which gave rise to four clusters with a resolution of 0.1 (**Figure 1E**). The clusters were annotated with CSF3R+ neutrophils (c0, 2, 3) and CSF1R+ macrophages (c1) (**Figure 1F** and **Supplementary Figure 2**). The MM samples demonstrated a significant increase in macrophages *versus* the MGUS (**Figure 1G**). Neutrophils from both MGUS and MM groups present TNFSF13B; however, macrophage TNFSF13B expression was enhanced significantly in MM samples (**Figure 1H**). Besides, we have detected a high heterogeneity of the neutrophils. Cluster 3 was detected exclusively in the MM samples. To clarify the features of this MM specialized neutrophil, we compared the gene expression between neutrophil cluster 3 and cluster 0 or cluster 2 and found that the MM specialized neutrophil present way higher level of DAMP signal-S100A9 (**Supplementary Figures 3A, B**).

**Figure 2:**
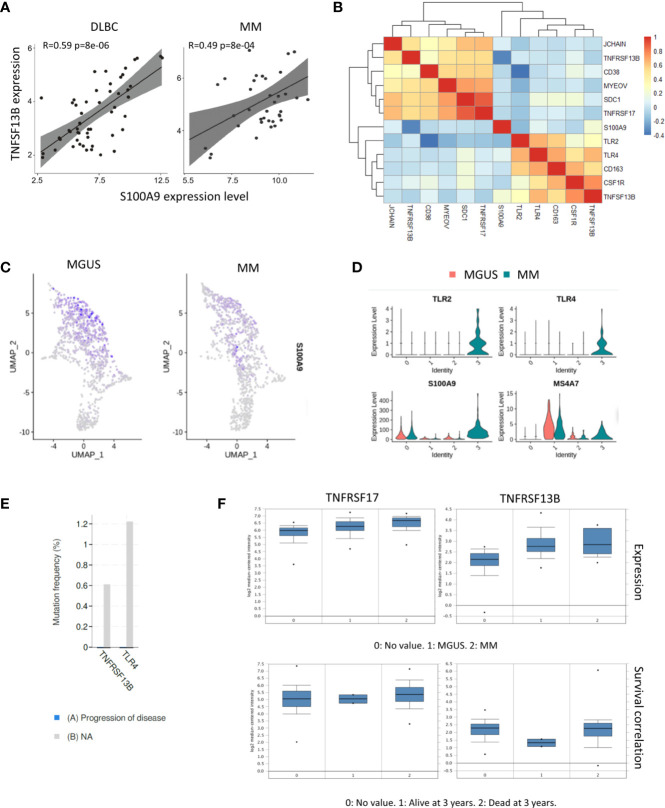
Correlation between TNFSF13B and S100A9 signal. **(A)** Scatter plots show the correlation between S100A9 and TNFSF13B expression in MM and DLBC samples. **(B)** Heatmap reveals the correlation between TNFSF13B related genes in myeloid cells and MM cells. **(C)** Feature plots show the projection of S100A9 expression onto the neutrophil and macrophages **(D)** Violin plots indicate the level of S100A9 expression in the neutrophil and macrophages. **(E)** In bar graphs, the progress of disease in the patient with TNFRSF13B and TLR4 mutant is quantified and compared. **(F)** Box plots demonstrate the correlation of TNFRSF13B and TNFRSF17 expression with MGUS and MM samples as well as their correlation with the 3-year survival rates.

Our previous study described that neutrophil derived S100A9 promotes TNFSF13B expression in the spleen. We analyzed the bulk RNA-seq dataset of samples from 74 MM patients (GSE104171) to explore the role of S100A9 in MM. The results demonstrated a clear correlation between S100A9 and TNFSF13B expressions. A tight correlation between S100A9 and TNFSF13B was also significant in the diffuse large B cell lymphoma (DLBC) samples, indicating that S100A9 might promote TNFSF13B expression in B cell-derived cells (**Figure 2A**). The correlation analyses for expressions of TNFSF13B and its receptors well-characterized their distinct functions and indicated a potential link between myeloma cells and macrophages. Specifically, TNFSF13B expression had close interactions with macrophage marker genes, namely, TLR and S100A9 genes; their receptor genes, TNFRSF13B and TNFRSF17, showed significant associations with the myeloma-related genes CD38, SDC1, and MYEOV (**Figure 2B**). The analysis of scRNA-seq data revealed neutrophils as a major source of S100A9 (**Figures 2C, D** **)**. To investigate whether TNFSF13B signals are involved in MM progression, we assessed potential correlations between TNFSF13B-related mutations using BioPortal (19). The results demonstrated that TNFRSF13B and TLR4 mutations might help slow malignant progression (**Figure 2E**). As for patient survival, we found that TNFRSF13B upregulation, instead of TNFRSF17, was associated with a lower 3-year survival rate in MM patients, ascribed to TNFRSF13B overexpression (**Figure 2F**). But S100A9 and TNFSF13B did not contribute to poor survival, which might be due to depleted MAM cells in advanced MM (data not shown). Nevertheless, the correlations of TNFRSF13B with myeloma-related genes and shorter survival, alongside TNFRSF13B mutations that thwarted MM advancement, indicate the importance of TNFSF13B signal in the MM prognosis of patients. The potential roles of S100A9 and TNFSF13B from MAM cells in MM progression also aroused our curiosity.

**Figure 3:**
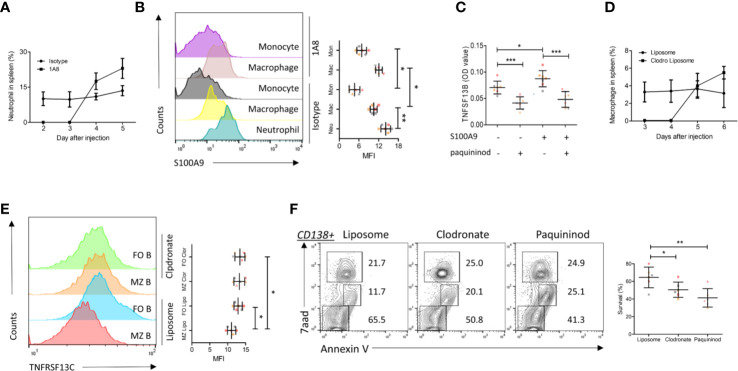
S100A9 and TNFSF13B contribute to the survival of plasma cell. **(A)** Neutrophils were depleted by injecting 1A8 monoclonal antibody. At the indicated time points, the proportion of CD45^+^Ly6G^high^ neutrophils in the spleen was measured by means of flow cytometry. **(B)** Flow cytometry histograms reveal the surface presence of S100A9 of splenic neutrophil, macrophage, and monocyte before and after neutrophil depletion. The data shown in the dot plot indicate the means ± SD of six mice from three independent experiments. **(C)** Isolated human monocytes were treated with S100A9 and/or paquininod, while the levels of TNFSF13B were measured 24 h after treatment. The data shown in the dot plot are the means ± SD of seven samples from three independent experiments. **(D)** Macrophages were depleted by injecting Liposomal Clodronate. At the indicated time points, the proportion of CD45^+^F4/80^+^ macrophages in the spleen was measured *via* flow cytometry. The data shown in the dot plot are the means ± SD of six mice from two independent experiments. **(E)** Flow cytometry histograms demonstrate the surface presence of TNFRSF13C of splenic MZ and FO B cell after macrophage depletion. **(F)** FACS plots indicate the proportion of 7aad^+^ dead and Annexin V^+^ apoptotic cells in the splenic CD138^+^ cells. The data shown in the dot plot are the means ± SD of six mice from three independent experiments. All data were analyzed through Student’s t- tests; *P < 0.05, **P < 0.01; ***P < 0.001.

As the above experiments have demonstrated that S100A9 in neutrophils (a major source) promoted TNFSF13B signals in MM patients, we examined the regulation of TNFSF13B by measuring the surface presence of S100A9 on splenic neutrophils, macrophages, and monocytes after neutrophil depletion in mice (**Figures 3A, B** **)**. The results showed that splenic macrophages and monocytes only expressed a low level of S100A9 in the steady state. S100A9 expression was upregulated after neutrophil depletion, suggesting that monocyte-derived cells could be an important source of both S100A9 and TNFSF13B (**Figure 3B**). We tested this hypothesis using human primary monocytes and observed a clear TNFSF13B expression. This expression could be triggered by exogenous S100A9 and blocked by the S100A9 inhibitor paquinimod (**Figure 3C**). We induced both neutrophil and macrophage depletion *via* injecting 1A8 mAb and clodronate liposomes for at least 3 days to validate TNFSF13B regulation *in vivo* (**Figure 3D**). As TNFSF13B induced the shedding of TNFRSF13C on mature B cells, we used TNFRSF13C surface expression on splenic B cells as an indicator of splenic TNFSF13B levels. As TNFSF13B and TNFRSF13C were identified in the marginal zone (MZ) near the red pulp, MZ B cells undergo stronger processing of TNFRSF13C shedding to lower TNFRSF13C surface expression *versus* follicular (FO) B cells. FACS histogram showed that neutrophil and macrophage depletion significantly enhanced TNFRSF13C surface expression on MZ B cells (**Figure 3E**), indicating a reduction of TNFSF13B expression in the splenic red pulp and thereby less TNFRSF13C shedding. Correlatively, a reduced survival rate of CD138+ cells was detected in the myeloid cell-depleted spleen, as supported by increased cell apoptosis after paquininod treatment (**Figure 3F**).

**Figure 4:**
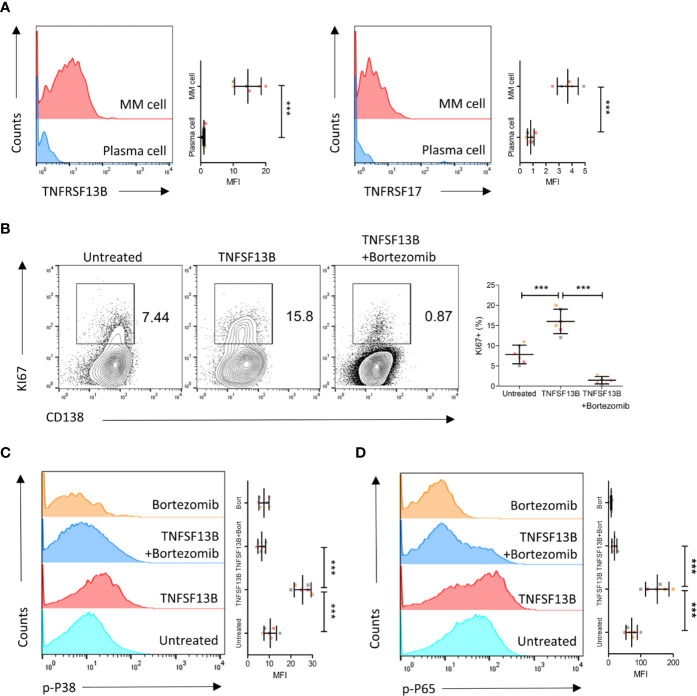
TNFSF13B/TNFRSF13B signal contributes to the canonical NF-*k*B signaling and the proliferation of MM cell. **(A)** Primary myeloma cells were derived from the patients with multiple myeloma. The expression of TNFRSF13B and TNFRSF17 on the myeloma cells. The data shown in the dot plot are the means ± SD of six samples from three independent experiments. **(B)** Myeloma cells were cultured with TNFSF13B either in the absence or presence of Bortezomib for 24 h. FACS plots indicate the proportion of KI67^+^ proliferative cells. The data shown in the dot plot are the means ± SD of six samples from three independent experiments. **(C)** The phosphorylation of p38 and **(D)** p65 has been demonstrated in FACS histogram after 30 min of treatment with TNFSF13B either in the presence or absence of Bortezomib. The data shown are the means ± SD of six samples from three independent experiments. ***P < 0.001.

We further validated these results in human primary myeloma cells isolated from MM patients. Both TNFRSF13B and TNFRSF17 surface expressions were upregulated on patient myeloma cells *versus* plasma cells from the healthy controls (**Figure 4A**). We subsequently assessed the efficacy of coculture of TNFSF13B with patient myeloma cells and significant pathways involved in the process. TNFSF13B markedly boosted myeloma cell proliferation, and this effect was suppressed after adding Bortezomib, a proteasome inhibitor (**Figure 4B**). It was reported in previous publications that TNFRSF13B transduces TNFSF13B signals *via* the canonical NF-*κ*B pathway, which requires the degradation of IkBa by the proteasome, while TNFSF13B–TNFRSF13C interaction stimulates the non-canonical NF-*κ*B pathway. To confirm whether the difference in NF-*κ*B signaling was present in MM, we determined p38 and p65 phosphorylation levels in myeloma cells treated with TNFSF13B, with or without Bortezomib, for 30 min. In the presence of Bortezomib, both p38 and p65 phosphorylation levels significantly decreased *versus* Bortezomib-free cells, indicating the involvement of the canonical NF-*κ*B pathway in both signaling pathways (**Figure 4C, D**). Taken together, the surface expressions of TNFSF13B receptors were upregulated on human myeloma cells, promoting myeloma cell proliferation and survival *via* TNFSF13B signal transduction through the canonical NF-*κ*B pathway.

## Discussion

In the current work, we upgraded the transcriptome assembly of bone marrow cells of MGUS and MM patients and revealed how myeloid-derived S100A9 boosted myeloid cell progression. Myeloid-derived S100A9 enhanced survival signals delivered by TNFSF13B receptors for myeloma cell survival and progression, resulting in shorter survival of MM patients within 3 years from diagnosis. In the animal experiments, we proved that S100A9 was required for both TNFSF13B production by myeloid cells and myeloma cell survival. Using primary myeloma cells of MM patients, we ascertained that TNFRSF13B transduced ligand signals to activate the canonical NF-*κ*B pathway, ultimately allowing cell proliferation.

MM ranks second among hematological malignancies regarding the prevalence rate. Even so, ascribed to its rare incidence across all tumor types, there are insufficient datasets of MM patients uploaded to TCGA. We integrated raw data of MGUS and MM patients from GEO, and pooled the data and conducted correlation analyses using Oncomine and cBioPortal. The integrated scRNA and bulk RNA-seq data covered a wider range of MM samples sufficient for gene expressing profile correlation analysis for prognosis prediction. Concerning the heterogeneity of myeloma cells among the patients, the integrated scRNA and bulk RNA-seq dataset can be used for identifying differentially expressed genes crucial for MM prognosis. And in contrast to typical bulk RNA-seq, scRNA-seq allows accurate discrimination between cell types for gene characterization and identification of tumor-associated cells.

The current work focused on the effects of surface gene expressions of TNFSF13B receptors on myeloma cell fate, particularly after the malfunction of neutrophils and macrophages in the MM microenvironment. Our results demonstrated that S100A9 administration upregulated TNFRSF13B and TNFRSF17 surface expressions on myeloma cells, indicating crosstalk between TLR activation and TNFSF13B signaling. In SLE, TLR signaling synergistically acts with TNFSF13B with the involvement of the TLR-associated signaling adaptor MyD88 (8). TLR activation by S100A9, alongside TLR–TNFSF13B interaction, cooperatively enhanced survival signals delivered to myeloma cells, consistent with the previous finding that TLR^+^ plasma cells showed higher level of autoantibodies against dsDNA in TNFSF13B-dependent lupus (22) (23).

In the steady state, S100A9 is constitutively expressed on neutrophils (24). Our results showed that myeloma-associated macrophages also expressed S100A9, which is affected by neutrophil depletion in lymph nodes (25). This observation agreed with other MM studies that dysregulated TLR stimulation may exacerbate malignant behaviors of plasma cells (26). The high number of tumor-associated neutrophils (TANs) and TAMs may increase the risk of poor survival of MM patients. In our animal experiments, the presence of neutrophils and macrophages resulted in less apoptosis in malignant plasma cells. This protection may be attributed to enhanced TNFSF13B signal transduction *via* S100A9/TLR binding, which has been proven to be critical for myeloma growth (14).

TNFSF13B is abundant in several cell types with close contact to B cells and myeloma cells, which indicates multiple sources of TNFSF13B signaling from neighboring cells. This process is done through TNFSF13B/TNFRSF13B or TNFRSF13C binding on B cells and TNFSF13B/TNFRSF13B or TNFRSF17 interaction on plasma cells (11). Our data indicate that TNFSF13B receptors TNFRSF13B and TNFRSF17 were expressed similarly on the MM cells for the signal transduction, while TNFRSF13C is undetectable. However, these receptors activate distinct downstream NF-*κ*B signaling pathways, as mentioned above, both contributing to MM maintenance (12, 27). Interestingly, our finding suggested the involvement of the proteasome inhibitor-Bortezomib in both canonical NF-*κ*B and MAPK/p38 signaling pathways, highlighting TNFRSF13B as a big player in S100A9-associated myeloma progression. Future studies can investigate how TNFRSF13B works for the canonical NF-*κ*B and MAPK/p38 stimulation and whether a TNFRSF13B-related gene signature can be used for prognosis prediction for MM patients.

In conclusion, myeloid-derived S100A9 can enhance TNFSF13B/TNFRSF13B expression on myeloma cells, stimulating both canonical and non-canonical NF-*κ*B pathways and thereby fuels tumor cell proliferation. TNFRSF13B expression on myeloma cells of MGUS and MM patients predicts a higher risk of poor prognosis. These findings suggest S100A9/TLR and TNFSF13B/TNFRSF13B as potential targets for anti-MM therapy.

## Data Availability Statement

The datasets presented in this study can be found in online repositories. The names of the repository/repositories and accession number(s) can be found in the article/**Supplementary Material**.

## Ethics Statement

The animal study was reviewed and approved by the School of Preclinical Medicine, Youjiang Medical University for Nationalities, Baise City, Guangxi Province, China.

## Author Contributions

LM and QT performed the *in vivo* experiments. JZ performed the *in vitro* experiments. ZW, LW, and QW prepared and assisted all the experiments. LY and SL supervised the bench work and contributed to the bioinformatic analysis. JS designed the experiments and performed the scRNA seq analysis. All authors contributed to the article and approved the submitted version.

## Funding

This research work was funded by the National Natural Science Foundation of China (#31970745).

## Conflict of Interest

The authors declare that the research was conducted in the absence of any commercial or financial relationships that could be construed as a potential conflict of interest.

## Supplementary Material

Supplementary Figure 1 Heat map annotated the top 10 differential gene expression of the nine main cell types in the bone marrow cells.

Supplementary Figure 2 Heat map annotated the top 10 differential gene expression of the nine main cell types in the myeloid cells.

Supplementary Figure 3 Volcano plots compared the differential gene expression between neutrophil subgroups. **(A)** Comparison between c0 and c3, **(B)** comparison between c2 and c3.
