# Supplementary figures and images for: S100A9 Derived From Myeloma Associated Myeloid Cells Promotes TNFSF13B/TNFRSF13B-Dependent Proliferation and Survival of Myeloma Cells
Source: Front Oncol. 2021 Jun 3;11:691705. doi: 10.3389/fonc.2021.691705 (PMC8210673; doi:10.3389/fonc.2021.691705)

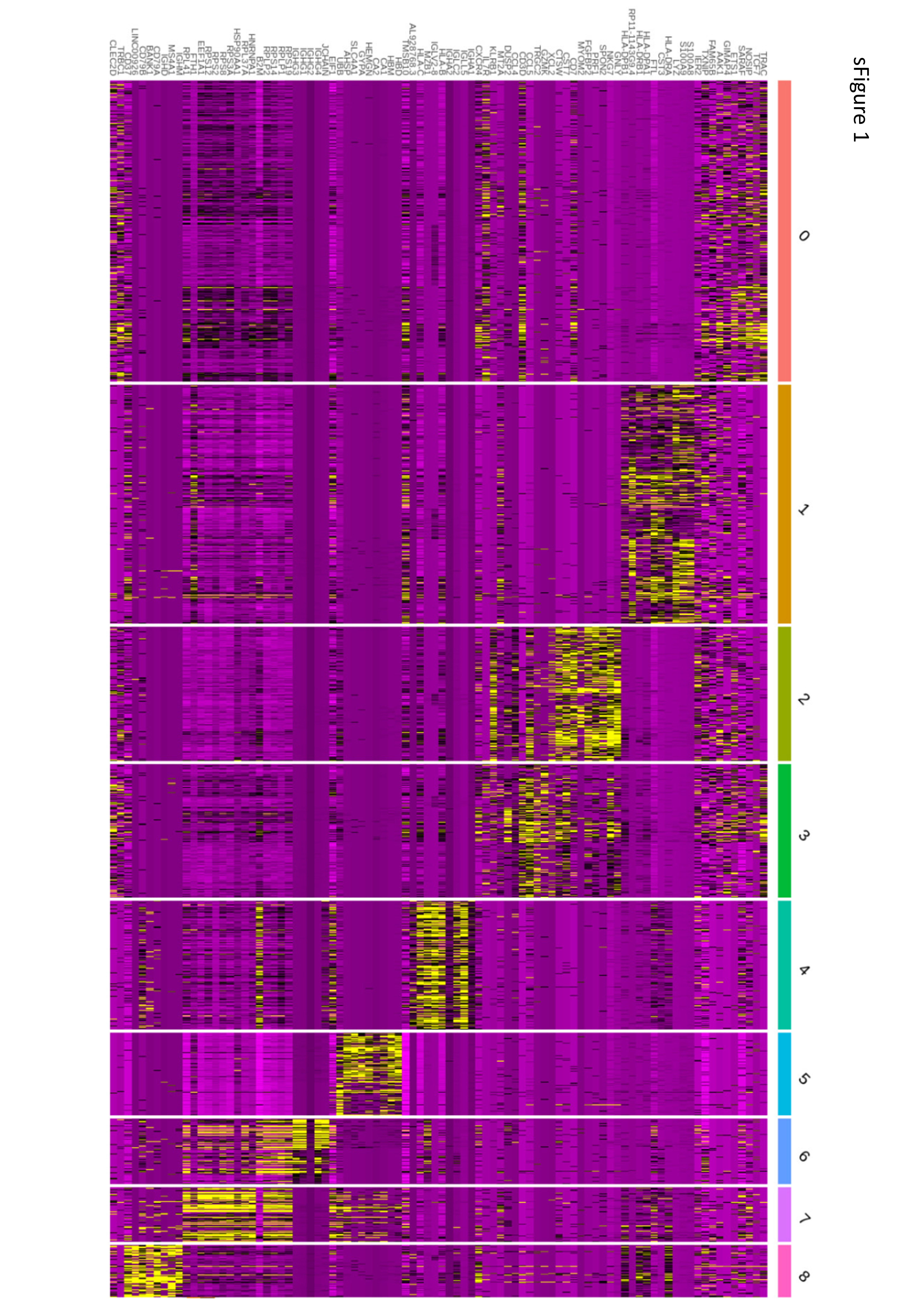

Supplement: Supplementary Figure 1 — Heat map annotated the top 10 differential gene expression of the nine main cell types in the bone marrow cells. [file Image_1.tif]

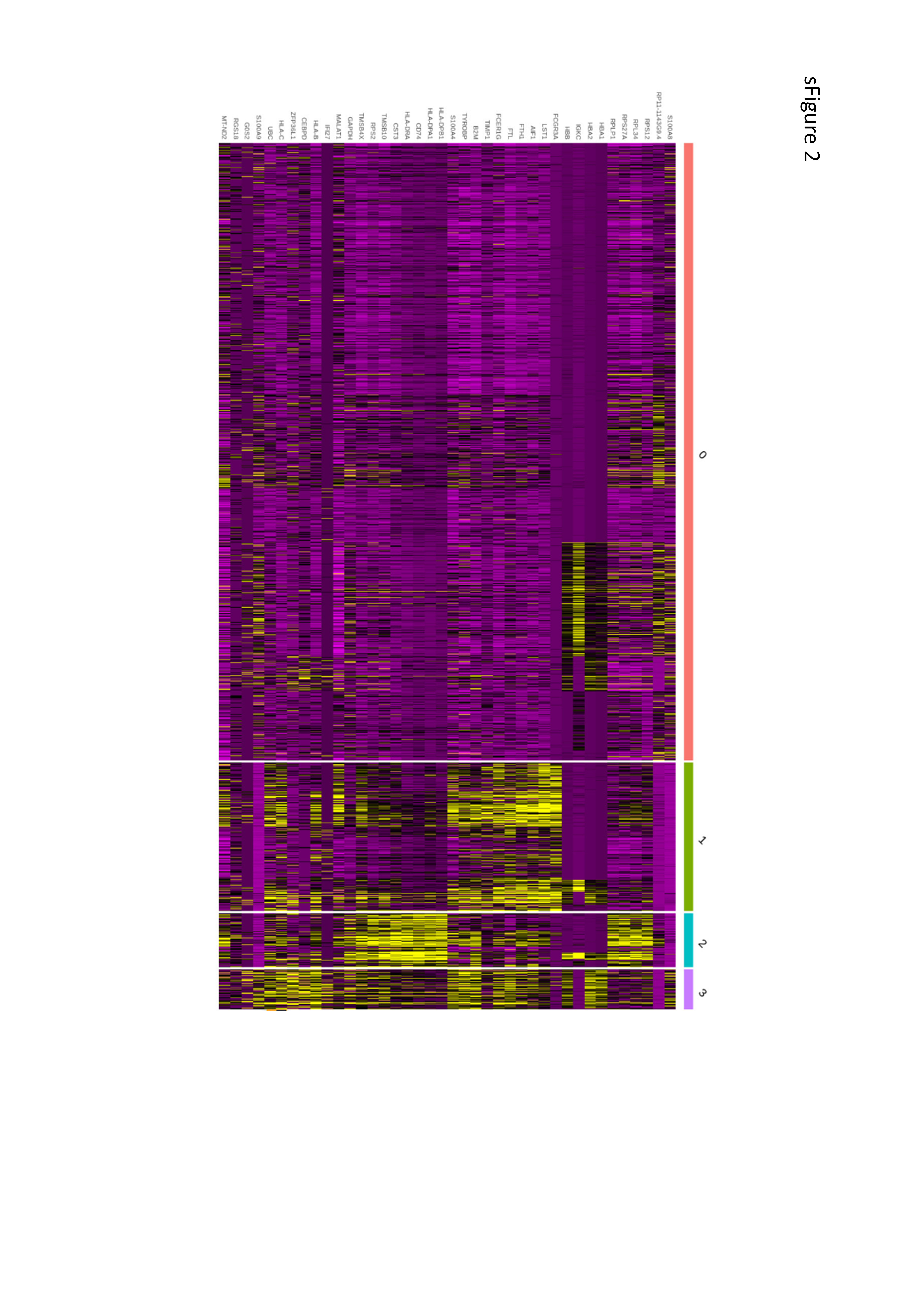

Supplement: Supplementary Figure 2 — Heat map annotated the top 10 differential gene expression of the nine main cell types in the myeloid cells. [file Image_2.tif]

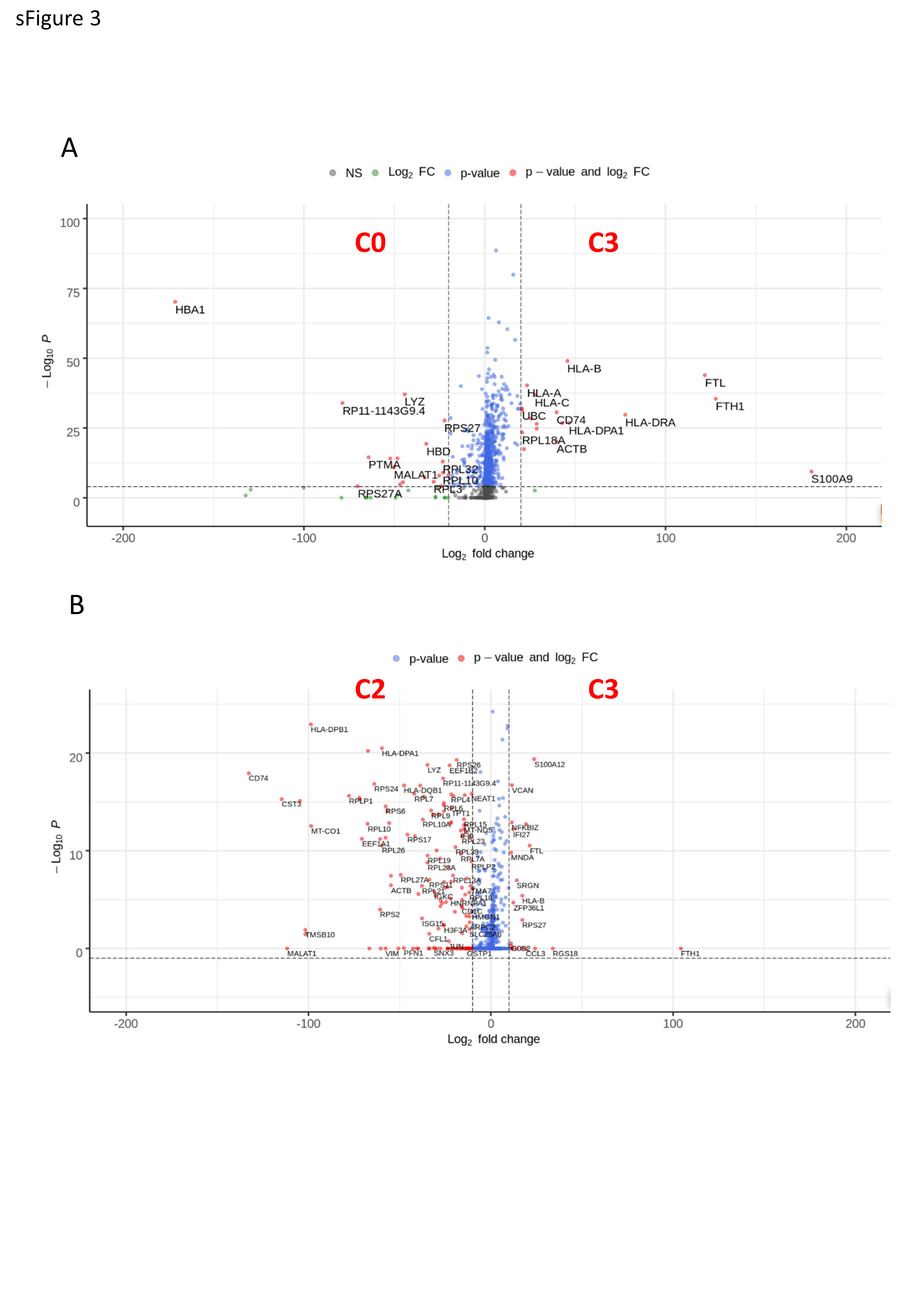

Supplement: Supplementary Figure 3 — Volcano plots compared the differential gene expression between neutrophil subgroups. (A) Comparison between c0 and c3, (B) comparison between c2 and c3. [file Image_3.tif]
